# The Role of Autonomous and Controlled Motivation in Exercise Intentions of Participants in a Mass Cycling Event

**DOI:** 10.3389/fpsyg.2017.00354

**Published:** 2017-03-16

**Authors:** Annick Willem, Jens De Rycke, Marc Theeboom

**Affiliations:** ^1^Movement and Sport Sciences, Ghent UniversityGhent, Belgium; ^2^Faculty of Physical Education and Physiotherapy, Vrije Universiteit BrusselBrussels, Belgium

**Keywords:** exercise, self-determination theory, motivation, mass participation event, road cycling

## Abstract

**Purpose:** This study used self-determination theory to examine the role of participants' autonomous and controlled motivation to exercise and to participate in a challenging mass cycling event and investigated whether the event enhanced intended and actual exercise behavior among the participants.

**Method:** Two hundred and twenty-eight subjects, having participated in the cycling event, completed a questionnaire shortly after the event and again 4 months later. The questionnaire measured self-reported cycling and exercise activity, training in preparation of the event, motivation to participate in the event, motivation to exercise, and future exercise intentions due to the event.

**Results:** Results showed that most participants were very active in cycling and other sports. The expected positive effect of autonomous motivation on exercise intentions and behavior could not be confirmed in our study. Multiple regression analyses revealed that the event had an enhancing effect on exercise intentions shortly after the event among participants that scored higher on controlled motivation to exercise (β = 0.15) and to participate (β = 0.15); also, participants were more satisfied with the event (β = 0.19) and had followed a preparation program before the event (β = 0.15). However, intentions and exercise behavior distinctively dropped 4 months after the event.

**Conclusions:** Events aiming to enhance their participants' exercise behavior need to attract less active participants and need to make additional efforts to prevent relapse in intentions and exercise behavior.

## Introduction

Motivation theory and need satisfaction can explain why people adhere to sustainable exercise behavior (Rhodes, 2014) because personal needs and motivations are related to different levels of commitment to exercise (Ryan and Patrick, 2009). Participants of mass participation sport events will not be amotivated to exercise, but different levels of motivation might be present among the participants, and the type of motivation might be very relevant in assessing whether mass participation in sporting events have a potential to enhance enduring exercise behavior. The popularity of mass participation sport events, combined with the large budgets and efforts organizers (i.e., often cities) spent on these events, have made researchers wonder whether such events act as “bread and circuses” or can be employed as drivers to increase sport participation and exercise among the population (Bauman et al., 2009). Tourism, sports, and recreation industries as well as hosting cities have already utilized the presumed exercise-enhancing effect of mass participation events as an important argument to promote events among funders, policy-makers, and the public (Murphy and Bauman, 2007; Coleman and Ramchandani, 2010). Several researchers have tried to find out whether such positive exercise effects are indeed present (Murphy and Bauman, 2007; Bauman et al., 2009; Lane et al., 2010, 2012; Funk et al., 2011; Ridinger et al., 2012; Stevinson and Hickson, 2013). These studies carefully confirm a potential positive effect of mass participation sport events on exercise behavior. However, studies are rarely longitudinal (Lane et al., 2012) and are therefore unable to measure long-term effects. Moreover, the effect of the event seems to depend on several factors, such as demographic factors, satisfaction with the event, prior exercise behavior, and motivation to participate (Funk et al., 2011). The demographic characteristics have been widely included in many studies, but participants' motivation has received far less attention (Funk et al., 2011), while motivation is key in enduring exercise engagements (Rhodes, 2014).

In particular, the self-determination theory (SDT) gained popularity to study the motivation to exercise (Fortier et al., 2012; Teixeira et al., 2012). SDT distinguishes among amotivation, autonomous, and controlled motivation. SDT also includes the basic psychological needs of autonomy, competence, and relatedness. Autonomous motivation is especially considered to be relevant for enduring exercise behavior (Titze et al., 2005). Autonomous regulated behavior refers to volition and meaningfulness of behavior without external pressure. It is considered to be related to more internalization and long-term change of behavior (Zerbinatti and Souitaris, 2005). In a systematic review of 66 empirical studies on SDT and exercise behavior, a positive relationship between intrinsic motivation and long-term exercise adherence was found (Teixeira et al., 2012). However, Teixeira et al. (2012) also mention that evidence for specific SDT constructs and exercise is yet unclear. Studies have focused on the conflict between autonomous and controlled motivation and the continuum from amotivation over extrinsic to intrinsic motivation (Deci and Ryan, 2000). Motivation can also explain extreme exercise behavior. For instance, in a study among adolescents, exercise dependency was related to the controlled dimensions of motivation (Downs et al., 2013).

In studying exercise behavior effects of mass participation sport events, two motivation processes are relevant, namely, motivation to prepare and to participate in the event and motivation to maintain exercise behavior (Funk et al., 2011). The latter thus refers to the motivation that affects whether the event participants will continue high levels of exercise in the post-event period compared to exercise intensity shortly before the event. Expressing post-event exercise behavior as a relative decrease compared to pre-event exercise is necessary because relapse periods after a short period of change in behavior (e.g., increased intensity of exercise) are very common (Sallis and Hovell, 1990; Kayser et al., 2014). In case of preparation for the event, a natural relapse effect after the event might be unavoidable. Derom et al. (2015) found that the average training period for a cycling event was 4 months and that this period increased depending on the distance cycled during the event. They found different types of cyclists depending on preparation and cycling distances. Also in the study of Derom et al. (2015), relapse was high even given the fact that almost all participants were very active cyclists. Considering this relapse effect and the importance of autonomous motivation for enduring exercise behavior (Sallis and Hovell, 1990; Titze et al., 2005; Kayser et al., 2014), the following hypothesis can be formulated:

Hypothesis 1a: The higher the autonomous motivation of the participants to exercise, the lower the relapse in exercise behavior.

Studies on the motives and motivation of preparing for and participating in mass sport participation events in relation to future exercise behavior are scarce. Funk (2008) explains that sport participants are consumers, consuming sport and sport events. This consumption can be explained to a large extent by sport consumer motivation (Funk et al., 2012). By participating in the event, a need is satisfied. This need can be physical, social, or personal. In the context of sport, the following motives are mentioned: socialization, performance, excitement, esteem, and diversion (Funk, 2008). Each of these can be a reason to participate in an event. In leisure studies, leisure motivation scales have been developed (e.g., the recreation experience preference scale) to list and categorize different motives for engaging in leisure activities, such as sport events (Manfredo et al., 1996). Studies indicate a relationship between motivation to participate and intentions to continue with the leisure activity, but this relationship is mediated by involvement—more involvement in the activity corresponds to a higher intention to continue (Kouthouris, 2009). Although, motivations to participate in the event might be very different from motivations to maintain exercise behavior, the two might be related. If more non-active people could be motivated to start training in preparation for an event and eventually become autonomously motivated to exercise, the event could serve as a real exercise promotion tool (Stevinson and Hickson, 2013). Hence, although exercising might start from participating in an event based on more controlled motives, this exercising might get internalized and move from more controlled to more autonomous motivated behavior (Ryan and Deci, 2000). A study on introjected regulation for exercise in adolescence showed that externally controlled exercise behavior might become internalized (Gillison et al., 2009). This was especially the case in girls. For internalization to occur, the event and exercise in preparation to the event need to give the participant a feeling of autonomy, competence, or relatedness (Kinnafick et al., 2014). Rosa et al. (2015), for instance, found that exercise can be a mean to achieve autonomy and self-cohesion. The motivational factors identified in the literature that enhance sport event participation (e.g., socialization, performance, and excitement) might fulfill these basic needs and be sources for autonomous motivation (Funk, 2008). Early and Corcoran (2013) studied the experience of 14 non-active people participating in running and walking events in Cambridge and confirmed that the events in their study had the potential to engage non-active people in exercise who experienced a sense of achievement, atmosphere, and relatedness. However, the event had a short-term perspective, namely, training and succeeding in completing the distance at the event, while maintaining exercise behavior has a more long-term perspective. There might be autonomous motivation for participation in an event, but when the event is finished, the goal is completed, and the need might be attained. Therefore, participants need to get attached and loyal to the sport itself and feel a kinship to, for instance, the cycling community (Funk, 2008). Vansteenkiste et al. (2004), for instance, showed that people framing an activity in a future goal, especially if this goal is intrinsically driven (e.g., being healthy), have greater exercise adherence. Participating in a challenging event might be part of a future goal to a healthier life.

Based on the insights from the scarce literature on the relationships between motivation to participate and exercise behavior in the context of a mass sport event (Titze et al., 2005; Funk et al., 2011; Early and Corcoran, 2013), the following hypothesis is formulated.

Hypothesis 2a: The higher the autonomous motivation of the participants to participate in the event, the lower the relapse in exercise behavior.

Funk et al. (2011) found that through a running event, a more positive attitude toward exercise occurred among the participants who were least active before participating in the event. These authors emphasize the problematic nature of studying the effects of a mass participation sport event, even with pre and post measures, and argue that behavioral assessments should be supported by attitudinal assessments. Changes in attitudes toward exercise can play a role in exercise behavior. According to the theory of planned behavior, people having a positive attitude toward exercise are more likely to change their behavior and become more active (Ajzen, 1991). Although, the behavioral change effect is relatively smaller than the change in intentions and several factors (e.g., behavioral control and habit formation) interfere in the intention-behavior relationship (Webb and Sheeran, 2006; Oliveira et al., 2014), an increase in intentions as well as in positive attitudes to exercise might result from participating in mass participation sport events, and this might trickle down to actual changes in exercise behavior. The meta-analysis of Hagger and Chatzisarantis (2009) showed that self-determined motivation precedes attitudes and intentions that in turn result (partly) in actual behavioral change. Hence, the formulated hypotheses are completed with exercise intention next to actual exercise behavior and the combined effect of motivation and intentions on behavior.

Hypothesis 1b: The higher the autonomous motivation of the participants to exercise, the higher the intentions to maintain exercise behavior.
Hypothesis 2b: The higher the autonomous motivation of the participants to participate in the event, the higher the intentions to maintain exercise behavior.
Hypothesis 1c: The higher the autonomous motivation of the participants to exercise, the stronger the positive relationship between intended and actual exercise behavior.
Hypothesis 2c: The higher the autonomous motivation of the participants to participate in the event, the stronger the positive relationship between intended and actual exercise behavior.

Farmanbar et al. (2013) explain that the complexity of making people physically active might require more than one theory to explain the change process, and they applied a combination of SDT and a transtheoretical model. According to the transtheoretical model, behavioral change is presumed to go through five stages of change, from being unaware of changing a specific behavior (e.g., exercise) to a complete behavioral change in which the behavior is fully routine practice (Prochaska and Diclemente, 1986; Webb and Sheeran, 2006). The relationship with motivation is that autonomous motivation will be higher in the maintenance stage and that autonomous motivation increases relative to controlled motivation when moving upward through the stages of change (Buckworth et al., 2007; Aspano Carron et al., 2016). The first stage is the precontemplation stage; in this stage, people are not partaking in exercise and are not interested in changing this in the next 6 months. It is expected that hardly any participants of mass participation sport events are in this stage. In the contemplation phase, intentions to start to exercise are developed. In the third stage, the preparation stage, people start planning to change their behavior, and in the action stage, these plans are executed. In the last stage, the maintenance stage, the changed behavior is maintained for at least 6 months. Participants of mass participation sport events will typically be found in this maintenance stage. However, if an event would succeed in activating non-active people, then participants in the action stage would participate and transfer to the maintenance stage due to the event. Participants starting to exercise a couple of months prior to the event to be able to participate in the event can be considered to be in the action stage when the event takes place. The event would then be a trigger to get into the action stage and a step toward the maintenance stage. Training might have the potential to bring participants in a higher stage of change from none or irregular exercise activity to more regular exercise activity. A specific training program might, therefore, be offered as a strategy to attract novice cyclists and to make them change their behavior. However, strategies on how to attract more people that are insufficiently active prior to training for the event are rarely suggested and even less studied. Therefore, we included in our study “following a preparation program,” “changes in stage of change,” and whether the latter is related to autonomous motivation. In particular, based on the literature (Ingledew and Markland, 2008), participants scoring high on autonomous motivation to exercise have a higher chance to be among the participants that evolved to a higher stage of change. A hypothesis is thus formulated on the relationship between autonomous motivation to exercise and changing exercise behavior based on the theory of change model (Prochaska and Diclemente, 1986; Webb and Sheeran, 2006).

Hypothesis 3: The higher the autonomous motivation to exercise of the participants that are not yet in the maintenance stage at the moment of the event, the higher the chance that they are in a higher stage of change 4 months after the event.

## Materials and methods

### Participants

Participants in this study were selected from an annual mass participation cycling event called the “Mon Ventoux.” The selected event received its popularity from an attractive and challenging goal, i.e., being able to climb by bike a well-known and steep mountain in France, the Mont Ventoux, but offered a training program to novice cyclists who were triggered by the popularity of cycling and cycling events but had not yet the ability and training condition to be active cyclists. The event consisted of five cycling tours with varying levels of difficulty. The more trained participants could choose to do one of the heavy cycling tours. In 2013, half of the participants cycled a tour that was limited to climbing the Mont Ventoux once (26 km) with the major challenge being succeeding in reaching the top on bike. Only this group is subject to this study.

The “Mon Ventoux” event was an annual cycling event organized by a Flemish recreational sports federation. Although, the event was abroad, it was only for Flemish participants and hence, a relatively small and local mass participation event. The event demanded a good physical condition, or if condition was poor, a preparation before one could participate. Participants could join the organized bus tour or could drive by car from their homes in Belgium to the venue in France, a neighboring country. The event was subsidized by the government and sponsored by companies allowing to keep the costs for the participants to a minimum. Several other cycling events existed in Belgium and the neighboring countries, but the “Mon Ventoux” event was unique because of the preparation program offered by the event organizer. Although, there were side activities, such as an after party in a nearby France village, the main objective of the event was a challenging cycling activity.

### Procedure

Data were collected via an on-line survey sent shortly after the event (T0) to all participants of the event and 4 months (T1) later to all respondents of the survey at T0. The follow-up survey allowed changes in intentions and actual exercise and cycling activity to be analyzed. A cover note accompanying the questionnaire provided details on why and by whom the research was executed, approval by the involved institutes, compliance with the ethical guidelines of the universities involved, assured confidentiality, and implied informed consent.

The online survey link was sent to all 2,460 participants. 662 participants completed the survey at T0, resulting in a response rate of 27%. At T1, all respondents included in the T0 sample received the link to the follow-up questionnaire. At T1, 476 participants completed the follow-up survey (72% response rate). Of these 476 respondents at T1, 455 could be combined with the data at T0. 49.9% of those were participants that cycled the heavy tours and were thus excluded from our study, resulting in a final sample of 228 respondents.

### Measures

#### Motivation

A modified version of the motivation scale used and tested several times in Flanders to measure motivation for running and cycling (Boen et al., 2009) and based on the Exercise Motivation Inventory (Markland and Ingledew, 1997) was used to measure whether participants were dominantly autonomously motivated or controlled motivated to participate in the event. Three items at T0—using a five-point Likert-scale ranging from 1 (strongly disagree) to 5 (strongly agree)—asked whether they participated “*for the competition element (price and rankings),” “because participating brings a certain amount of prestige,”* and “*because other people would look up to me”* (controlled motivation). The Cronbach alpha value for this scale was 0.69. Seven items (Cronbach alpha of 0.71) measured at T0 the autonomous motivation to participate. The items included the following: *I participated “to relax,” “for positive health benefits,” “because I like challenges,” “to live a unique experience,” “because of the inspiring location,” “for personal development,” “to enjoy the social aspect.”* Exploratory factor analysis resulted in two factors that clearly separated the three controlled motivational items from the other seven items with factor loadings on one of the two factors ranging between 0.5 and 0.8. *Exercise motivation* was measured using the Dutch version of the behavioral regulation exercise questionnaire (BREQ2) based on the self-determination theory (Mullen et al., 1997; Markland and Tobin, 2004; De Meester et al., 2014). Fifteen items measured the two ground motivation forms, controlled and autonomous motivation, on a five point Likert scale at T0. Cronbach alpha values indicated a sufficient reliable scale with 0.86 and 0.69 at T0 for autonomous and controlled motivation, respectively.

#### Exercise behavior and intentions

For exercise, a distinction was made between cycling and one main other exercise that participants were doing. To calculate the average hours per month in the year before the event (T0) of cycling and of practicing one other exercise, the average time per week or month was asked at T0 and the number of months per year one was practicing these sports. At T1, the average hours of cycling and one other exercise in the fourth month after the event (T1) were asked in the survey. To measure future intentions to exercise due to participating in the event, questions were asked at T0 and T1 about the extent to which the event was perceived as having a stimulating effect on exercise. These items were based on the future exercise intention measurement in the work of Funk et al. (2011), but they were adapted to the context of the specific cycling event that also intended to attract novice cyclists. At T0, seven items were given and scaled on a five point Likert-scale: “*I train harder in preparation of the event,” “After the event, I maintain intense training,” “I was stimulated to join cycling clubs or groups,” “The event stimulated me to exercise more,” “The event stimulated me to exercise throughout the year,” “Without the event, I would exercise less,” and “I have trained more because of the preparation days and preparation program.”* The six items at T1 were “*I train harder because of the event,” “After the event, I train more than before,” “I was stimulated to join cycling clubs or groups,” “The event stimulated me to exercise more,” “The event stimulated me to exercise throughout the year,” and “Without the event, I would exercise less*.” The average score of these items resulted in a score on the variable “intentions” (Cronbach alpha was 0.7 at T0 and 0.64 at T1).

#### Stages of change

To study in which stage of physical activity the respondents belonged, we asked at T0 and T1 whether the respondents were physically active involving increased heartbeat and sweating for at least 3 to 5 times a week during 20–60 minutes according to the international standard for minimum level of physical activity per week and based on the stages of change and the trans-theoretical model of Prochaska and Diclemente (1986) as well as whether they had the intention to become sufficiently active (Marcus et al., 1992). In particular, we asked respondents whether they were physically active during more or less than 6 months and, if not, if they had the intention to change this in the next month, 6 months or later (Marcus et al., 1992).

#### Event preparation program

Respondents were asked whether they had increased their hours of cycling in preparation of the event and, if so, with how many hours of cycling. Participants were also asked if they followed the preparation program offered by the event organizer.

#### Other control variables

Gender was included as a control variable because literature has indicated that motivation differs between males and females in relation to exercise behavior (Guerin et al., 2012). Respondents were asked whether cycling was their main exercise activity and how many years they were cycling as exercise activity. We also asked whether it was their first cycling event in which they participated and whether they cycled the heavy tour. Satisfaction with the event was assessed by asking for satisfaction with the location, price/quality, atmosphere, experience, and the cycling itself. In addition, respondents gave a score out of 10 to rate their overall satisfaction. These scores were combined to calculate an average satisfaction score per respondent.

### Analysis

First, a missing-values analysis using SPSS was done but did not indicate patterns in missing data. Missing values were further handled in the regression analyses as excluding cases, listwise. Second, linear regression analyses in SPSS were used to test our hypotheses and study relationships between the control variables and the dependent variables intentions and average hours of exercise. The longitudinal data allowed us to study the effects of the independent variables on the intentions at T1, hours of cycling at T1, and changes in average hours of cycling between T0 and T1. Conditions for regression analysis, namely, linearity and constant variance, independency, and normal distribution of the error terms, were tested and found sufficient. Hence, no transformation was needed for the variables used in the models. However, residualized change scores were calculated by regressing average hours of cycling at T1 on these variable at T0. These residualized change scores were used in the regression analysis. Interaction effects were tested for the interactions between intentions and autonomous motivations to participate and to exercise in relation to the dependent variable change in hours of cycling between T0 and T1 in order to analyze whether intentions were reinforced by motivation. In the first regression models, only the control and dependent variables were added. In the second step, the motivation variables were added. In the third step, the interaction effect was entered, and improvements in the model were assessed by changes in adjusted R^2^. In addition, the interaction effect was also tested using the PROCESS macro in SPSS of Hayes (2009). Third, multinominal logistic regression analysis in SPSS was used to regress change in stage of change on the hours of exercise and motivation variables. The dependent variable in this regression analysis consisted of the categories “a lower stage of change at T1 compared to T0” and “a higher stage of change at T1 compared to T0” with “no change in stage of change” as the reference category.

## Results

The sample consisted of 75% males. The mean hours of cycling in the year before the event, including extra preparation for the cycling event, by the participants in our sample was 25.73 h per month. The mean number of months they were cycling ranged between 9 and 10 months a year. There was a decrease in mean hours of cycling per month at T1 compared to T0 with 12.77 h. 11.3% cycled for less than a year, 85.1% of the participants considered cycling as their main exercise activity, and the mean number of years of cycling was 7.21. In our sample, 53.6% practiced other types of exercises as well. 48.7% participated for the first time in a cycling event. In general, participants were highly satisfied with the event as was indicated by the mean score on satisfaction with the event of 9 on 10. Some of the participants (31.6%) regularly cycled and indicated that the event did not require special training for them. A small percentage (3.1) indicated that they did not regularly cycle but also did not prepare for the event. The others (65.4%) prepared for the event by increasing the intensity of cycling. 67.5% of the participants in our sample participated in at least one preparation training tour or workshop organized by the event organizers. 29.4% of the participants indicated that they were not physically active according to the physical activity norm during the last 6 months. Descriptive statistics and bivariate correlations of the main variables are presented in Table 1.

**Table 1:** **Bivariate correlations and descriptive statistics**.

	**M**	**SD**	**5**	**6**	**7**	**8**	**9**	**10**	**11**
1. Average hours of cycling at T0	25.73	16.98							
2. Average hours of cycling at T1	12.96	13.91							
3. Average hours of other sport at T0	5.71	8.63							
4. Average hours of other sport at T1	6.34	9.83							
5. Change in average hours of cycling									
6. Change in average hours of other sport			−0.09						
7. Intention to exercise at T0	2.95	0.64	−0.04	0.09					
8. Intention to exercise at T1	2.38	0.077	0.13^*^	0.03	0.50^**^				
9. Autonomous motivation to participate at T0	4.18	0.53	−0.06	0.00	0.30^**^	0.15^*^			
10. Controlled motivation to participate at T0	2.39	0.97	0.02	−0.03	0.18^**^	0.13^*^	0.34^**^		
11. Autonomous motivation to exercise at T0	4.35	0.58	0.00	0.01	0.17^**^	0.04	0.36^**^	0.03	
12. Controlled motivation to exercise at T0	1.38	0.55	0.05	−0.07	0.21^**^	0.18^**^	0.17^*^	0.31^**^	0.07

** p < 0.01;

* *p < 0.05*.

Results of the multivariate linear regression analysis with event-based intention to exercise are displayed in Table 2. In the first model, only the control variables are included. These explain 17% of the variance in event-based intention to exercise at T0. Satisfaction with the event and following the preparation program were especially significantly related to the intention variable at T0. In the second model, the four motivation variables were included, and a moderate effect size was found. Both controlled motivation to participate and to exercise were positively related to intention at T0 and contributed significantly to the model. Intentions measured at T1 were not related to the same variables. Only having cycling as main exercise was positively related to intentions.

**Table 2:** **Multivariate regression analysis on the contribution of motivation to event-based intention to exercise**.

**Dependent variables**	**Intentions T0**				**Intentions T1**			
	**Model 1**		**Model 2**		**Model 1**		**Model 2**	
**Independent and control variables**	**β(SE)**	**95% CI**	**β (SE)**	**95% CI**	**β (SE)**	**95% CI**	**β (SE)**	**95% CI**
Gender	0.12(0.09)	−0.017,0.355	0.08(0.09)	−0.069,0.297	0.13(0.13)	−0.013,0.480	0.11(0.13)	−0.059,0.44
Average hours of cycling T0	−0.07(<0.00)	−0.008,0.003	−0.15(<0.00)	−0.011,0.00	−0.13(<0.00)	−0.013,0.002	−0.17(<0.00)	−0.016,0.00
Average hours of other sport T0	−0.05(0.01)	−0.013,0.006	−0.07(0.01)	−0.014,0.005	−0.06(0.01)	−0.018,0.008	−0.07(0.01)	−0.019,0.007
Satisfaction with event	0.19(0.09)^***^	0.091,0.442	0.13(0.10)	−0.028,0.383	0.06(0.12)	−0.124,0.341	0.04(0.14)	−0.203,0.357
Cycling as main exercise	0.12(0.12)	−0.034,0.455	0.012(0.12)	−0.021,0.453	0.18(0.16)^*^	0.062,0.710	0.18(0.16)^*^	0.079,0.725
Years of cycling	−0.05(<0.00)	−0.012,0.006	−0.03(<0.00)	−0.010,0.007	−0.01(0.01)	−0.012,0.010	<0.00(0.01)	−0.012,0.011
First time event	0.04(0.08)	−0.110,0.212	0.08(0.08)	−0.066,0.251	−0.02(0.11)	−0.240,0.186	0.01(0.11)	−0.204,0.228
Average hours of preparation	0.12(0.00)	−0.001,0.009	0.07(<0.00)	−0.003,0.008	−0.10(<0.00)	−0.011,0.003	−013(<0.00)	−0.013,0.001
Preparation program	0.15(0.09)^*^	0.029,0.376	0.15(0.09)^*^	0.026,0.364	0.02(0.12)	−0.193,0.267	0.01(0.12)	−0.210,0.252
Autonomous motivation to participate			0.11(0.10)	−0.057,0.320			0.04(0.13)	−0.195,0.319
Controlled motivation to participate			0.15(0.05)^*^	0.007,0.183			0.11(0.06)	−0.036,0.204
Autonomous motivation to exercise			0.02(0.08)	−0.126,0.169			−0.01(0.10)	−0.210,0.193
Controlled motivation to exercise			0.15(0.08)^*^	0.012,0.310			0.12(0.10)	−0.031,0.375
R^2^	0.17		0.25		0.07		0.11	
ΔR^2^			0.08				0.04	
N	219		219		219		219	
F(df1,df2)	4.74(9,210)^***^		5.22(4,206)^***^		1.71(9,210)		1.87(4,206)^*^	

*** p < 0.001;

* *p < 0.05*.

Results of the regression with change in average hours of cycling as a dependent variable are displayed in Table 3. People that had cycling as their most important form of exercise had the smallest drop in average hours of cycling compared to people that did not have cycling as their main sport. This was the only significant relationship found in this analysis. The predictors included in the model could not explain the variance in changes in average hours of cycling. This was also the case for changes in average hours of cycling and one other sport. None of the predictors were significant in the models. Intentions to exercise at T0 could not predict changes in exercise at T1. The interaction effects of intentions and autonomous motivation to exercise could also not predict changes in exercise behavior. The effect size for these analyses were low.

**Table 3:** **Multivariate regression analysis on the contribution of motivation to changes in cycling T1–T0**.

**Dependent variables**	**Residuals cycling T1-T0**							
	**Model 1**		**Model 2**		**Model 3**		**Model 4**	
**Independent and control variables**	**β (SE)**	**95% CI**	**β (SE)**	**95% CI**	**β (SE)**	**95% CI**	**β (SE)**	**95% CI**
Gender	−0.06(2.11)	−5.944,2.382	−0.05(2.19)	−5.863,2.770	−0.05(2.15)	−5.857,2.633	−0.05(2.014)	−5.804,2.647
Average hours of cycling T0	0.01(0.06)	−0.118,0.135	−0.01(0.07)	−0.141,0.129	0.01(0.06)	−0.119,0.135	0.01(0.06)	−0.119,0.134
Average hours of other sport T0	−0.01(0.11)	−0.240,0.197	−0.01(0.11)	−0.246,0.204	−0.01(0.11)	−0.238,0.200	−0.02(0.11)	−0.242,0.194
Satisfaction with event	−0.02(1.99)	−4.464,3.388	0.03(2.46)	−3.853,5.852	−0.01(2.11)	−4.405,3.893	<−0.00(2.17)	−4.317,4.233
Cycling as main exercise	0.17(2.78)^*^	1.043,11.985	0.19(2.84)^*^	1.526,12.741	0.18(2.80)^*^	1.131,12.168	0.18(2.80)	1.192,12.233
Years of cycling	0.08(0.10)	−0.079,0.304	0.08(0.10)	−0.098,0.300	0.08(0.10)	−0.079,0.305	0.08(0.09)	−0.086,0.299
First time event	−0.07(1.82)	−5.496,1.692	−0.07(1.89)	−5.509,1.954	−0.07(1.83)	−5.516,1.688	−0.07(1.83)	−5.478,1.723
Average hours of preparation	−0.15(0.06)	−0.227,0.006	−0.16(0.06)	−0.235,0.004	−0.15(0.06)	−0.225,0.009	−0.14(0.06)	−0.224,0.010
Preparation program	0.13(1.97)	−0.273,7.499	0.12(2.04)	−4.91,7.547	0.13(1.99)	−0.211,7.629	0.13(1.99)	−0.191,7.627
Autonomous motivation to participate			−0.08(2.25)	−6.371,2.518				
Controlled motivation to participate			0.06(1.06)	−1.325,2.850				
Autonomous motivation to exercise			−0.02(1.76)	−3.880,3.045				
Controlled motivation to exercise			0.04(1.79)	−2.506,4.563				
Intentions at T0			−0.04(1.64)	−3.994,2.453				
Intentions at T0xautonomous motivation to participate							−0.04(0.29)	−0.741,0.403
Intentions at T0xautonomous motivation to exercise					−0.03(0.30)	−0.707,0.457		
R^2^	0.095		0.102		0.096		0.096	
ΔR^2^			0.008		0.001		0.001	
N	219		219		219		219	
F(df1,df2)	2.444(9,210)		1.67(5,205)		2.208(1,209)		2.226(1,209)	

* *p < 0.05*.

In the multinominal logistic regression with a lower or higher stage of change as categories and no change in stage of change as the reference category of the dependent variable, results indicated that the hours of preparation and hours spent on the other or second sport were important (see Table 4). The more average hours of other sport at T0, the lower the chance that a participant would have changed stages of change (either to a lower or higher stage of change). The more hours of preparation for the event, the lower the chance to find the participant in a higher stage of change after the event. However, both effects were rather small. An effect was also notable for the predictor motivation to participate. The higher the score in the variable controlled motivation to participate, the lower the chance to find the participant in a lower stage of change after the event compared to the stage of change at the event.

**Table 4:** **Multinominal Logistic regression analysis on changes in stages of change**.

	**Lower level in stage of change**		**Higher level in stage of change**	
	**Exp(B), SE**	**CI**	**Exp(B), SE**	**CI**
Average hours of second sport at T0	0.925^**^, 0.027	0.878,0.975	0.930^*^,0.035	0.868,0.997
Average hours of cycling at T0	0.990, 0.013	0.966,1.015	0.948^*^, 0.025	0.902,0.997
Average hours of preparation	1.004, 0.010	0.983,1.024	0.943^**^, 0.023	0.901,0.987
Autonomous motivation to exercise	1.131, 0.318	0.607,2.111	0.990, 0.426	0.429,2.284
Controlled motivation to exercise	1.405, 0.317	0.755,2.612	1.649, 0.400	0.753,3.613
Autonomous motivation to participate	1.332, 0.365	0.651,2.722	0.896, 0.441	0.378,2.0125
Controlled motivation to participate	0.678^*^, 0.197	0.461,0.999	0.695, 0.247	0.429,1.127
Nagelkerke	0.165			
Chi-square	33.767^**^			

** p < 0.01;

* *p < 0.05*.

## Discussion

Based on previous studies on motivation and exercise (Hagger and Chatzisarantis, 2009), higher autonomous motivation to exercise is related to higher intentions to maintain exercise behavior. Hence, applied in the present study, autonomous motivated participants in the event were assumed to show higher levels of intentions to maintain exercise due to the stimulating effect of participating in the event. However, the relationship between the motivation variables and intention to exercise variable was surprising. Participants indicating high levels of controlled motivation to exercise scored high on intentions to maintain future exercise. Hypothesis 1b on a positive relationships between autonomous motivation and exercise intentions is thus rejected. At first sight, this contradicts the literature, saying that autonomous motivation is important (Frederick and Ryan, 1995; Pedersen, 2002; Teixeira et al., 2012). However, this controlled motivation did not result in behavioral change, confirming the literature that controlled motivation to exercise is not a good basis for enduring exercise adherence (Wilson et al., 2008). Hypothesis 1a on the relationship between autonomous motivation and exercise behavior is thus also rejected because neither high controlled nor high autonomous motivation were related to low relapse in exercise behavior. There was also no evidence for the interaction effect of autonomous motivation and intentions on exercise behavior (Hypothesis 1c).

The findings are thus partly in line with the literature. However, the lack of evidence on an enhancing effect of autonomous motivation on behavioral change in our data is disappointing and contradicts previous research where, for instance, less autonomous motivated female runners regressed more from running compared to more autonomous motivated (Titze et al., 2005). Participating in a mass participation sport event might be too related to the controlled side of exercise motivation. This is also confirmed by the fact that controlled motivation to participate was positively related to intentions to maintain exercise measured at T0, rejecting Hypothesis 2b. Participating in such an event is prestigious and very visible to friends and family. Having participated in such an event creates the expectation to maintain high levels of cycling. Participants that perceive such external pressures might be more inclined to higher future exercise intentions. For some people, the controlled motivational factors might thus be important and might even impact intention to exercise. The event itself can thus be considered as a controlling factor in motivation. It might even be that people who need external pressure to maintain their exercise activity were deliberately searching for challenging events and were therefore participating in the event. It is also possible that some already active participants need the event to be motivated to intensify their exercise activity in preparation for a challenging event and to persist in their cycling activity over a longer period of time. This is in line with the finding that extreme exercise dependency can be based on controlled motivation (Downs et al., 2013). Controlled motivational factors might thus play a role in attracting people to the event and might be used to attract low-active people. In combination with a post-event program, exercise intentions might be positively changed even if the initial intentions to maintain exercise behavior were based on controlled motivation. However, Hypotheses 2a and 2c were not confirmed because controlled motivation to participate did not affect the magnitude of relapse in exercise behavior. Hence, again this effect of controlled motivation is not helpful for long-term exercise behavior. According to Thøgersen-Ntoumani and Ntoumanis (2006), controlled motivation might even have negative effects on exercise behavior on the long-term, e.g., when the external pressure results in social physique anxiety.

Webb and Sheeran (2006), in their review on intention-behavioral relationship studies, have pointed to several reasons why intentions did not result in behavioral change in the studies. Lack of longitudinal data was one of the reasons; however, that is not applicable here. Another reason might be that the behavior is not sufficient under control of the respondents. However, exercise behavior is considered as one the behavioral types that is largely under control of the individual. Kouthouris (2009) found that involvement with the particular sport was important for intentions to maintain in practicing the sport. Our data shows that intentions at T1 and lower relapse were only related to having cycling as the main exercise. Hence, being dedicated to cycling as one's main central leisure activity seems to be the dominating independent variable explaining the variance in exercise behavior change, and this variable might be related to “involvement,” a variable used in leisure studies (Kouthouris, 2009). Involvement is, however, a multidimensional concept, but only centrality and attraction were found to be related to intentions in the study of Kouthouris (2009). The dimension of attraction in the involvement scale refers to pleasure and fun one perceives from the activity or exercise and is thus related to autonomous motivation factors. Hence, the variable “cycling as main exercise” might have served in our sample as a proxy for leisure involvement and absorbing effects of autonomous motivation. Scores on autonomous motivation were on average very high in our sample confirming the fact that most of the participants in the event were already heavy cyclists that might have fully internalized cycling in their autonomous regulated behavior.

A number of other reasons for the lack of sustained positive intentions to maintain exercise and behavioral change might be found in the characteristics of the event participants. Participants in the cycling event were very active in cycling, which they had been doing for many years and some even in cycling competition, confirming previous results in Flanders, Belgium (Derom et al., 2015). Although, the participants were cycling a lot, this did not mean that they were participating frequently in cycling events. One third of the participants participated for the first time in a cycling event. Many of the participants were, thus, already very active in cycling before they decided to participate in a cycling event instead of the cycling event being the start of a cycling career. Similar results were found in the literature (Bowles et al., 2006; Funk et al., 2011). The fact that the event required a foreign trip (from Belgium to the south of France) might also be an important reason why only people very engaged in cycling were willing to do this effort (Funk et al., 2007). The majority prepared for the event but in addition to already intensive cycling habits. Cycling was for many participants so high in the months prior to the event that a decrease was unavoidable. The regression to the mean phenomenon might have played here. Only strong habits, here cycling habits (Webb and Sheeran, 2006) or as already mentioned strong involvement (Kouthouris, 2009), can prevent relapse.

The theory of planned behavior can also provide insight into the steps from intentions to actual behavioral change (Prochaska and Diclemente, 1986). The purpose of the study was to see whether a mass participation event can serve as an exercise promotion intervention. Therefore, stages of change were measured for regular exercise according to health norms. One third of the participants in our sample did not position themselves in the maintenance stage of change. Hence, although most were active cyclists, they were not physically active enough according to the international health norm. This could be due to the fact that cycling was often too much concentrated on 1 day per week, too irregularly practiced, or only regularly practiced in preparation of an event. Hence, although the percentage of low-active participants (only 3.1%) at the moment of the event was low, the percentage of participants that need to increase their physical activity level to reach the norm was much larger. Furthermore, there were some unexpected relationships between changes in stages of change of the participants and the variables measured. The more average hours participants spent on a sport other than cycling, the lower the chance that the participant would have changed stages of change (either to a lower or higher stage of change). Thus, participants for which cycling was not central in their exercise behavior because they are merely occupied with another sport had less chance that the cycling event changed their exercise behavior. An assumption derived from this is that mass participation events might only have an impact on the intensity of practicing the sport of the event and not on exercise in general. Hence, when events are used as exercise promotion, the sport at the event should be a sport that can be easily practiced on a regular basis because no spill-over effects on other types of exercise might exist.

The relationship with motivation again showed an unexpected link. Hypothesis 3 is rejected because autonomous motivation to participate or to exercise did not result in a positive evolution of the participants toward a higher stage of change. On the contrary, the more controlled motivation to participate, the less chance to be in a lower stage of change 4 months after the event. This again confirms that controlled motivation might be important for mass participation event participants. Being more controlled motivated was also no reason to experience a higher relapse effect. Internalized external or social pressures (introjected motivation) turned out to be important in previous research for physical activity and exercise among youngsters but might not be helpful in motivating people to adhere to long-term exercise behavior because the behavior is not fully internalized (Dishman et al., 2015). This might be the reason why we do not find an effect on exercise behavior itself but only on intentions.

The results on theory of change also confirmed the relapse effect because 25% of the participants were found in a lower stage of change 4 months after the event. The hours of preparation for the event did not help to bring participants to a higher stage of change. On the contrary, the more hours of preparation, the lower the chance to find these participants in a higher stage of change. In general, it can be concluded that the training program as well as the controlled motivation had a “bubble” effect. Participants were motivated to participate, trained for the event through extra hours of cycling, and followed the preparation program, but after the event, a large relapse occurred regardless of the type of motivation.

## Limitations and future research

There are a number of limitations situated in the particular setting when drawing conclusions on the potential of mass sport events and exercise behavior change. The event was far too challenging to motivate sedentary people to become active unless a long and medically controlled preparation program was offered. Hence, the preparation program might have not been sufficient. It must also be emphasized that cycling intentions and behavior are not constant, and it remained unclear to what extent effects maintain for periods of more than 4 months. Seasonal effects might have been at play as well, although cycling was not indicated by the respondents as a seasonal activity because on average participants cycled 10 months a year. Numerous other factors are at play (e.g., climate) that can temporary reduce cycling intensity. Finally, a self-selection bias typical in mail survey studies might have occurred, for instance, with the risk that more autonomous motivated people were participating in the study.

The event was challenging, and therefore no generalizations to less demanding participation sport events (e.g., long distance walking or short distance running) can be made. The event studied required a financial and time effort of the participants, excluding more disadvantaged groups, and people from a lower social-economic status are more at risk for low levels of exercise; hence, our results cannot be generalized to all groups in society. Our study has also limitations in terms of the period studied, the single case-study design, and the lack of a measurement point a period before the event. Furthermore, dairies or step counters could be used to control for the self-reported data as well as including a control group of non-participants. However, these kinds of research approaches are difficult to realize. Nonetheless, real-life events as such provide interesting insights that are the basis for further in-depth study.

Previous studies have shown that the motivation-intention-behavior relationship is complex and might be moderated and mediated with a number of other factors. Hence, future research might include other variables, such as habitual cycling behavior, goal attainment (cfr. work of Vansteenkiste et al., 2004), and involvement. For instance given the high involvement of the participants in cycling behavior in our sample it might be interesting to study the relationship between the different dimensions of involvement and the motivation types in relation to exercise adherence further developing the work of Kouthouris (2009).

## Conclusions

Our study adds to the existing literature on motivation theory and exercise by including motivation to exercise, motivation to participate, intensity of cycling prior to the event, effect of following a preparation program, and by measuring not only intentions but also actual behavior change after a 4-month period of participation in a mass cycling event. Results are, however, not that promising: respondents clearly indicated positive intentions to exercise more due to the event but with only limited effects on behavioral change. All relationships found with intention to exercise are short term. Only for the participants having cycling as their main sport were intentions put in practice because this group had a smaller drop in hours of cycling. The aspiration of the event organizer was to attract individuals who engage in low levels of physical activity to a cycling challenge, and to support these individuals via a preparation program, with a view of promoting their long-term participation in physical activity. However, the findings of the study suggest this aspiration was not realized. The number of low-activity people that could be inspired by the event to change their exercise behavior and become more active is low. As the literature has already indicated, changing exercise behavior for a longer period requires autonomous motivation to exercise (Ingledew and Markland, 2008), and a mass cycling event might not be the ideal setting to strengthen autonomous motivation to exercise.

## Ethics statement

Ethical Committee Ghent University Hospital (UZ Ghent). A cover note accompanying the questionnaire provided details on why and by whom the research was executed, approval by the involved institutes, compliance with the ethical guidelines of the universities involved, assured confidentiality, and implied informed consent.

## Author contributions

AW contributed to the development of the study design, the questionnaire design, statistical analysis, interpretation of the results and drafting the manuscript. JD collected the data and contributed to the data analysis. MT contributed to the study design and critical review of the manuscript. All authors read and approved the final manuscript.

## Funding

This study was supported by the Policy Research Centre on Sports and was partly funded by the Flemish Government.

### Conflict of interest statement

The authors declare that the research was conducted in the absence of any commercial or financial relationships that could be construed as a potential conflict of interest.
